# Difference in the functional connectivity of the dorsolateral prefrontal cortex between smokers with nicotine dependence and individuals with internet gaming disorder

**DOI:** 10.1186/s12868-017-0375-y

**Published:** 2017-07-27

**Authors:** Xin Ge, Yawen Sun, Xu Han, Yao Wang, Weina Ding, Mengqiu Cao, Yasong Du, Jianrong Xu, Yan Zhou

**Affiliations:** 1 Department of Radiology, Ren Ji Hospital, School of Medicine, Shanghai Jiao Tong University, Shanghai, 200127 People’s Republic of China; 2 Department of Child & Adolescent Psychiatry, Shanghai Mental Health Center, Shanghai Jiao Tong University, Shanghai, 200030 People’s Republic of China

**Keywords:** Functional magnetic resonance imaging, Internet gaming disorder, Nicotine dependence, Resting-state functional connectivity, Dorsolateral prefrontal cortex

## Abstract

**Background:**

It has been reported that internet gaming disorder (IGD) and smokers with nicotine dependence (SND) share clinical characteristics, such as over-engagement despite negative consequences and cravings. This study is to investigate the alterations in the resting-state functional connectivity (rsFC) of the dorsolateral prefrontal cortex (DLPFC) observed in SND and IGD. In this study, 27 IGD, 29 SND, and 33 healthy controls (HC) underwent a resting-state functional magnetic resonance imaging (rs-fMRI) scan. DLPFC connectivity was determined in all participates by investigating the synchronized low-frequency fMRI signal fluctuations using a temporal seed-based correlation method.

**Results:**

Compared with the HC group, the IGD and SND groups showed decreased rsFC with DLPFC in the right insula and left inferior frontal gyrus with DLPFC. Compared with SND group, the IGD subjects exhibited increased rsFC in the left inferior temporal gyrus and right inferior orbital frontal gyrus and decreased rsFC in the right middle occipital gyrus, supramarginal gyrus, and cuneus with DLPFC.

**Conclusion:**

Our results confirmed that SND and IGD share similar neural mechanisms related to craving and impulsive inhibitions. The significant difference in rsFC with DLPFC between the IGD and SND subjects may be attributed to the visual and auditory stimulation generated by long-term internet gaming.

## Background

Internet gaming disorder (IGD), also known as problematic internet use, is the excessive and recurrent use of online internet games [1]. IGD is different from substance abuse or drug addiction such that no substance or chemical intake is involved; however, excessive internet use may lead to physical dependence similar to that observed in other addictions [2]. Currently, IGD has become a serious mental health issue around the world, thereby requiring additional investigation, as exemplified by its inclusion as a condition for further study in Section 3 of the Diagnostic and Statistical Manual of Mental Disorders (5th Edition, DSM-5) [3]. The following diagnostic criteria for IGD were suggested: time distortion, time spent longer than initially intended and planned time, use of internet activity to cope with or escape problems, compulsive behavior, deception about the extent of use, failure to stop or control use, and preoccupation with internet use when offline [4–6]. Notably, many of these behavioral symptoms resemble substance-related disorders [7–9].

Currently the precise pathogenesis of IGD remains unclear. A few studies suggested that the risk factor of IGD is related to the increased prevalence of substance dependence [10–12]. Numerous studies found that IGD and substance dependence shared similar neural mechanisms, such as nicotine dependence [9, 13, 14]. On the basis of behavioral addiction, researchers have been attempting to associate IGD with other behavioral problems that can lead to addiction, such as drug abuse, alcohol abuse, and nicotine dependence [7, 15]. Our previous study revealed that smokers with IGD exhibited decreased resting-state functional connectivity (rsFC) in the right rectus gyrus and increased rsFC in the left middle frontal gyrus with post cingulate cortex (PCC), compared with nonsmokers with IGD. Furthermore, negatively correlation was found in the PCC connectivity with the right rectus gyrus with Chen’s internet addiction score (CIAS) of smokers with IGD before correction. The results suggested that, compared with the nonsmokers with IGD, smokers with IGD had alterations of function in brain regions related to executive motivation and function [9]. However, Vergara et al. [16] delineated a general pattern of hypoconnectivity in the precuneus, insula, postcentral gyrus, and visual cortex of substance consumers. In addition, connectivity reduction between postcentral and one resting state networks covering right fusiform and lingual gyri showed their significant association with severity of hazardous drinking. In smokers, hypoconnectivity between the thalamus and putamen was observed. By contrast, the angular gyrus showed hyper-connectivity with the precuneus linked to smoking and significantly correlated with the severity of nicotine dependence. These results suggested that particular effects of alcohol and nicotine can be separated and identified. Han et al. [8] found IGD subjects and alcohol dependence (AD) have positive rsFC values in the dorsolateral prefrontal cortex (DLPFC) and cingulate, cerebellum, as well as negative rsFC values between the DLPFC and orbitofrontal cortex. The AD group was found to have positive rsFC values between the DLPFC, striatal areas, and temporal lobe, whereas the IGD group shows negative rsFC values among these areas. They concluded that the both groups may have deficits in executive function.

In this study, we attempted to detect the difference between the rsFC of individuals with IGD and those of smokers with nicotine dependence (SND), and explore the mechanism of this difference. According to Han et al. [8], cravings induced by particular substances such as alcohol are closely associated with DLPFC activity [17]. Furthermore, DLPFC is thought to play key roles in mediating clinical symptoms of executive dysfunction, alcohol dependence, including impulsivity, and aggravation of abuse potential [18]. The present study aims to assess DLPFC-seeded rsFC in IGD and SND.

## Methods

### Participants

The current study was approved by the Research Ethics Committee of Ren Ji Hospital and School of Medicine, Shanghai Jiao Tong University, China No.[2016]079k(2) with written informed consent from all subjects. All participants were informed of the aims of our study before MRI examination. Of the 86 participants included in the study and were evaluated by brain MRI from Jan 2016 to Dec 2016, 27 had IGD, 29 SND, and 30 healthy controls (HC). As described in our previously study [9], the IGD subjects who met the diagnostic questionnaire for internet addiction (i.e., YDQ) test modified by Beard and Wolf [19] were recruited from the psychological outpatient clinic at the Shanghai Mental Health Center. While, the SND and HC groups were recruited through advertisements. The IGD group played internet game approximately 42–70 h (mean ± SD: 44.31 ± 10.27) per week. The appropriate questions from the Structured Clinical Interview for DSM-IV [20] was used to assess nicotine dependence. The participant from the IGD and HC groups had never smoked, and no participant self-reported daily alcohol consumption or other substance use disorder (SUD). All the SND subjects began smoking 2–10 years before the current study onset. They are all daily smokers, and they smoke approximately 10–45 cigarettes (mean ± SD: 21 ± 1.76) per day. CIAS [21], self-rating anxiety scale (SAS) [22], self-rating depression scale (SDS) [23], Barratt impulsiveness scale-11 (BIS-11) [24], and Fagerstrom test of nicotine dependence (FTND) [25] were performed to assess the clinical characteristics of the participants. CIAS is a self-reported measure with good reliability and validity and has been used to measure the severity of internet addiction [26]. The FTND is a six-item self-report questionnaire used to assess the severity of nicotine dependence [25]. All questionnaires were initially written in English and then translated into Chinese.

All the participants were right handed, and none of participants had (1) previous hospitalization for a history of major psychiatric disorders or psychiatric disorders; (2) a substance use disorders other than nicotine addiction; (3) mental retardation; (4) neurological illness or injury; (5) intolerance to MRI.

### MRI acquisition

Images were obtained using a 3.0T MRI scanner (GE Signa HDxt 3T, USA) with a standard head coil. Restraining foam pads was used to reduce head motion and earplugs were used to reduce scanner noise. The SND group was required to abstain from smoking 1 h before scanning. Resting-state functional MRI data were acquired using a gradient-echo echo-planar sequence as described in our previously study [9]. Afterward, 34 transverse slices (repetition time [TR] = 2000 ms, echo time [TE] = 30 ms; field of view [FOV] = 230 × 230 mm^2^; 3.6 × 3.6 × 4 mm^3^ voxel size) were obtained aligned along the anterior commissure-posterior commissure line. Each fMRI scan lasted 440 s. During the scanning, the participants were instructed to stay awake with their eyes closed and do not think any specific subjects. After scanning, the subjects were asked to confirm they remain awake during the scan. In addition, high-resolution T1-weighted anatomical images (TR = 6.1 ms, TE = 2.8 ms, TI = 450 ms, slice thickness = 1 mm, gap = 0, flip angle = 15°, FOV = 256 × 256 mm^2^, number of slices = 166, 1 × 1 × 1 mm^3^ voxel size) using a 3D fast spoiled gradient recalled sequence images.

### Statistical analysis

The demographic and clinical measures of the groups were compared. One-way ANOVA tests were carried out using Statistical Package for the Social Sciences software (version 18) to assess the differences among the 3 groups. Bonferroni post hoc tests were then performed to assess the differences between each pair of groups. A 2-tailed p value of 0.05 was considered statistically significant for all analyses.

Functional MRI preprocessing was performed using a toolbox for data processing and analysis for brain imaging (http://rfmri.org/dpabi) [27]. After discarding the first 10 volumes of each functional time series, the remaining 210 images were preprocessed. Slice-timing correction, realignment, and spatial normalization, as well as smoothing (6 mm full width at half maximum), were conducted. Nuisance covariates, including time-series predictors for global, cerebrospinal fluid, white matter and six movement parameters were regressed out to improve the signal-to-noise ratio and minimize the motion artifact. No participant in this study exhibited movement greater than 1.5 mm with maximum translation in *x*, *y*, or *z*, axes or maximum rotation of 1.5° in the 3 axes. Moreover, the mean framewise displacement (FD) was computed by averaging the FDi of each subject from each time point [28]. No difference among the mean FD values of the groups (p = 0.71). Then, we applied temporal filtering (0.01–0.08 Hz) to the time series of each voxel to reduce the influence of high-frequency noise and low-frequency drift [29–32]. DLPFC was used as the region of interest (ROI) seed in the current study, and the DLPFC template was made as described in previous research [8].

Then, the blood-oxygen-level-dependent signal time series of the in each voxel within the seed region were averaged to generate the reference time series. A correlation map for each subject was produced by computing the correlation coefficients between the reference time series and time series from the other brain voxels. Z values were converted from the correlation coefficients by Fisher’s z-transform to improve the normality of the distribution [31]. Afterwards, the individual z-scores were entered into SPM8 for the one-sample *t* test in a voxel-wise manner, which was performed to determine the brain regions with significant positive or negative correlation with the DLPFC within each group. Individual scores were entered into SPM8 for random effect analysis, and then one-way ANOVA were performed.

Differences with regard to age, sex, education, SAS scores, SDS scores, and BIS-11 scores were regressed for each rsFC along the subject dimension. Multiple comparison corrections were performed using the AlphaSim program in the Analysis of Functional Neuroimages (AFNI) software package (NIMH, Bethesda, MD USA; available at http://afni.nimh.nih.gov/afni) [33], as determined by Monte Carlo simulations. Significant differences were defined as those which survived a threshold of p < 0.05, AlphaSim corrected (a combined threshold of p < 0.001 for each voxel and a cluster size >11 voxels, yielding a corrected threshold of p < 0.05). Group interaction analyses were then carried out with two-sample t-tests. The differences were obtained according to the results of ANOVA by applying the mask to limit the t-tests to the significant brain areas. AlphaSim corrected threshold p < 0.05 (a combined threshold of p < 0.001 and a cluster size >11 voxels) was performed as multiple comparison correction. Brain regions exhibiting significant differences were then masked on the MNI brain templates.

## Results

### Demographic and clinical characteristics

Table 1 listed the demographic and clinical measures for each group. No significant difference was observed between the IGD and HC groups in terms of age and years of education. However, significant differences were found between the IGD and SND groups and between the HC and SND groups. Difference with respect to sex was obtained because no female smoker participated in the study. The IGD subjects had higher CIAS, SAS, SDS, and BIS-11 compared with other 2 groups.

**Table 1:** Demographic and clinical characteristics of the three groups

	IGD (n = 27)	HC (n = 33)	SND (n = 29)	F value (p value)	p1-2	p1-3	p2-3
	(Mean ± SD)	(Mean ± SD)	(Mean ± SD)				
Age (years)	20.78 ± 2.20	20.78 ± 2.51	22.58 ± 2.41	5.56 (0.005)	1	0.02	0.01
Sex (F/M)	8/19	6/27	0/33	12.091 (0.001)	0.30	0.001	0.02
Education (years)	11.26 ± 1.67	12.67 ± 2.58	13.03 ± 2.06	5.19 (0.007)	0.43	0.01	1
Chen Internet Addiction Scale (CIAS)	75.15 ± 9.95	44.27 ± 9.81	46.76 ± 8.34	98.29 (<0.001)	<0.001	<0.001	0.45
Self-Rating Anxiety Scale (SAS)	51.33 ± 10.16	39.61 ± 6.39	44.48 ± 8.96	14.09 (<0.001)	<0.001	0.01	0.08
Self-rating depression scale (SDS)	54.56 ± 10.80	43.12 ± 8.85	47.44 ± 9.13	10.66 (<0.001)	<0.001	0.02	0.24
Barratt Impulsiveness Scale-11 (BIS-11)	61.22 ± 8.44	52.82 ± 6.64	52.41 ± 7.50	12.36 (<0.001)	<0.001	<0.001	1
FTND			6.52 ± 2.11				

p 1-2 for IGD group versus HC group, p 1-3 for IGD group versus SND group, p 2-3 for HC group versus SND group

*SD* standard deviation, *HC* healthy control, *IGD* internet gaming disorder, *SND* smokers with nicotine dependence, *FTND* Fagerstrom test of nicotine dependence

### DLPFC connectivity analysis

#### One-way ANOVA analysis in three groups

**Fig. 1:**
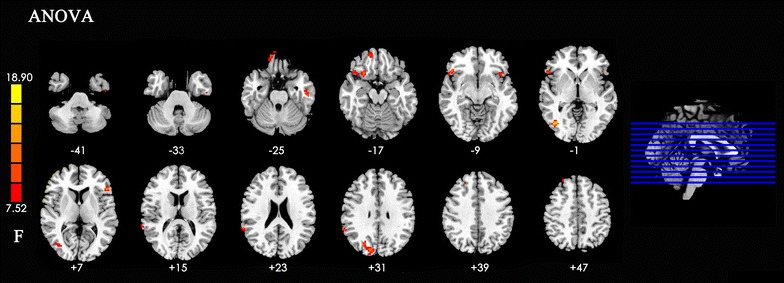
Significant differences in functional connectivity of different brain regions with DLPFC changes among the three groups. *Note*: The *left part of figure* represents the participant’s right side, and *right* represents the participant’s left side. *DLPFC* dorsolateral prefrontal cortex

Significant differences were observed among the rsFC with the DLPFC in the left side of inferior temporal gyrus, insula, inferior frontal gyrus, right side of the middle temporal gyrus, supramarginal gyrus, cuneus, superior orbital frontal gyrus, insula, inferior orbital frontal gyrus, and superior frontal gyrus (Table 2; Fig. 1).

**Table 2:** Significant differences in functional connectivity of different brain regions with DLPFC changes among the three groups

	Peak MNI coordinate region	Peak MNI coordinates			Number of cluster voxels	Peak F value
		x	y	z		
1	Left inferior temporal gyrus (BA20)	−51	−15	−27	29	15.69
2	Left insula (BA48)	−36	16	−11	20	8.46
3	Left inferior frontal gyrus (BA45)	−51	27	6	22	14.70
4	Right middle temporal gyrus (BA19)	42	−72	0	47	21.28
5	Right supramarginal gyrus (BA40)	63	−48	27	30	11.32
6	Right cuneus (BA19)	15	−84	30	75	13.43
7	Right superior orbital frontal gyrus (BA11)	15	54	−21	39	13.96
8	Right insula (BA47)	27	21	−18	22	13.61
9	Right inferior orbital frontal gyrus (BA38)	48	27	−12	61	10.56
10	Right superior frontal gyrus (BA9)	24	42	42	15	9.68

*MNI* Montreal Neurological Institute, *BA* Brodmann’s area, *DLPFC* dorsolateral prefrontal cortex

*p* < 0.05, AlphaSim-corrected

#### Between-group analysis of DLPFC connectivity: IGD versus HC

**Fig. 2:**
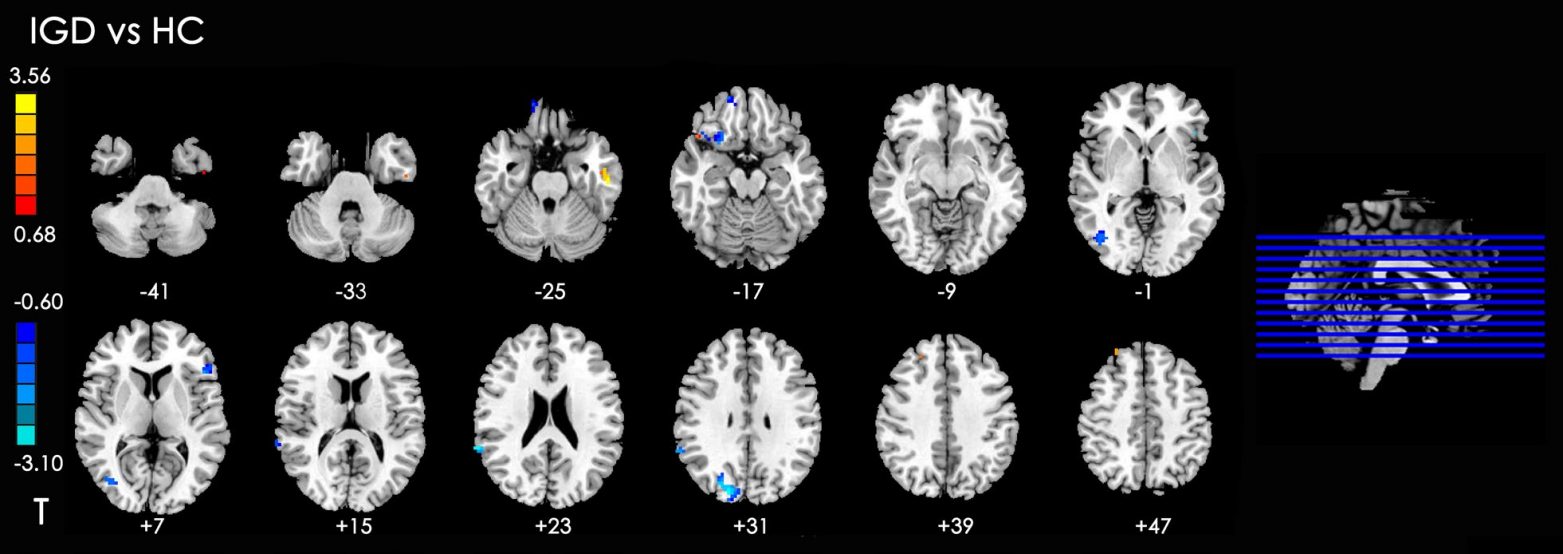
Significant between-group differences in functional connectivity of different brain regions with DLPFC between the IDG with HC subjects. The *t*-score bars are shown on the *left*. *Red* indicates IGD > HC, and *blue* indicates IDG < HC. *Note*: The *left part of figure* represents the participant’s right side, and *right* represents the participant’s left side. *DLPFC* dorsolateral prefrontal cortex, *IGD* internet gaming disorder, *HC* healthy control

The IGD group exhibited significantly increased rsFC in left inferior temporal gyrus, right superior temporal gyrus, and right middle frontal gyrus with the DLPFC, compared with the HC group. In addition, decreased rsFC was found in the left inferior frontal lobe, right side of the medial frontal orbital gyrus, insula, middle occipital gyrus, superior temporal gyrus, and cuneus with the DLPFC (Table 3; Fig. 2).

**Table 3:** Summary of functional connectivity with DLPFC changes in IGD compared with the HC group

	Peak MNI coordinate region	Peak MNI coordinates			Number of cluster voxels	Peak *t* value
		x	y	z		
1	Left inferior temporal gyrus (BA20)	−54	−21	−27	29	4.14
2	Right superior temporal gyrus (BA38)	48	24	−21	14	2.51
3	Right middle frontal gyrus (BA9)	27	42	42	15	3.15
4	Left inferior frontal gyrus (BA45)	−45	24	0	22	−2.81
5	Right medial frontal orbital lobe (BA11)	18	57	−18	16	−1.88
6	Right insula (BA48)	27	15	−18	21	−2.22
7	Right middle occipital gyrus (BA19)	39	−75	3	47	−2.26
8	Right superior temporal gyrus (BA22)	65	−45	24	30	−3.62
9	Right cuneus (BA19)	18	−84	27	67	−3.67

*t* > 0 indicates IGD group >HC group in functional connectivity, *t* < 0 indicates IGD group <HC group in functional connectivity with DLPFC

*MNI* Montreal Neurological Institute, *BA* Brodmann’s area, *DLPFC* dorsolateral prefrontal cortex, *IGD* internet gaming disorder, *HC* healthy control

p < 0.05, AlphaSim-corrected

#### Between-group analysis of DLPFC connectivity: SND versus HC

**Fig. 3:**
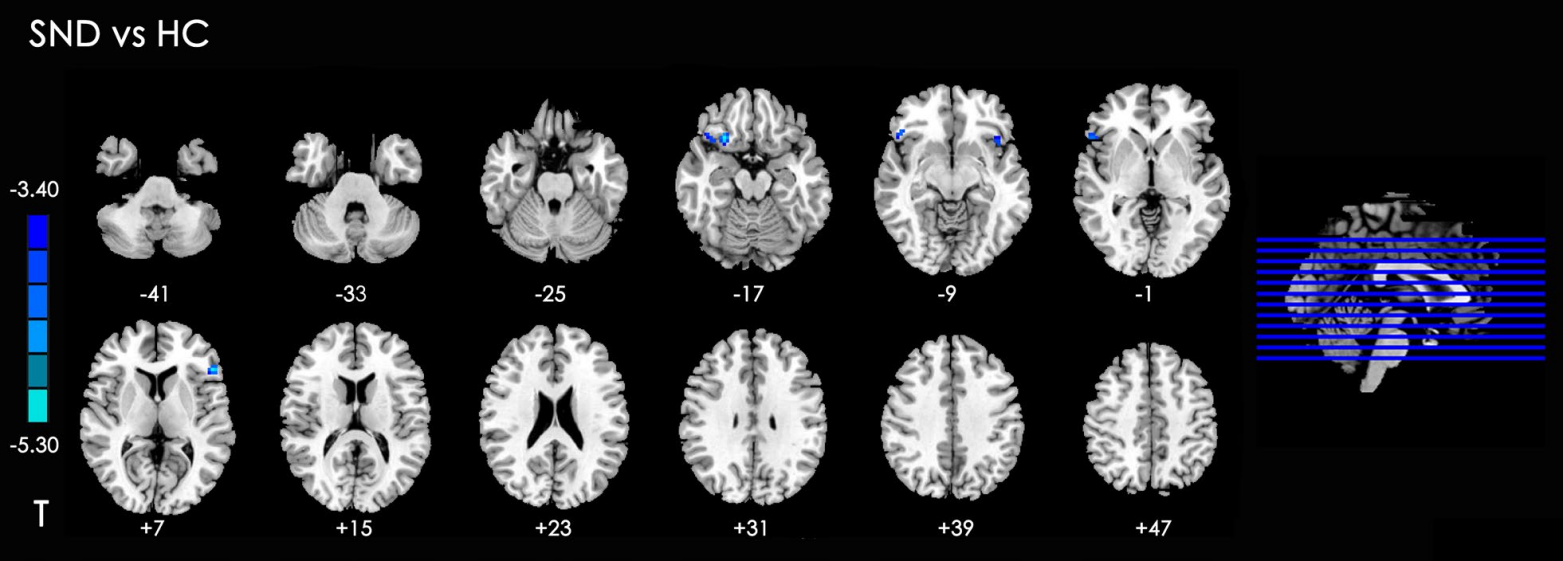
Significant between-group differences in functional connectivity of different brain regions with DLPFC between SND and HC subjects. The t-score bar is shown on the *left*. *Blue* indicates SND group < HC. *Note*: The *left part of figure* represents the participant’s right side, and *right* represents the participant’s left side. *DLPFC* dorsolateral prefrontal cortex, *SND* smokers with nicotine dependence, *HC* healthy control

The SND group showed significantly decreased rsFC in bilateral insula, left inferior frontal gyrus, and right inferior orbital frontal gyrus with the DLPFC (Table 4; Fig. 3).

**Table 4:** Summary of functional connectivity with DLPFC changes in SND group compared with the HC group

	Peak MNI coordinate region	Peak MNI coordinates			Number of cluster voxels	Peak *t* value
		x	y	z		
1	Left inferior frontal gyrus (BA45)	−51	27	6	20	−5.47
2	Left insula (BA48)	−42	15	−9	13	−4.10
3	Right insula (BA47)	27	21	−18	21	−5.41
4	Right inferior orbital frontal gyrus (BA38)	48	27	−12	27	−5.70

*t* < 0 indicates SND group <HC group in functional connectivity with DLPFC

*MNI* Montreal Neurological Institute, *BA* Brodmann’s area, *DLPFC* dorsolateral prefrontal cortex, *SND* smokers with nicotine dependence, *HC* healthy control

*p* < 0.05, AlphaSim-corrected

#### Between-group analysis of DLPFC connectivity: IGD versus SND

**Fig. 4:**
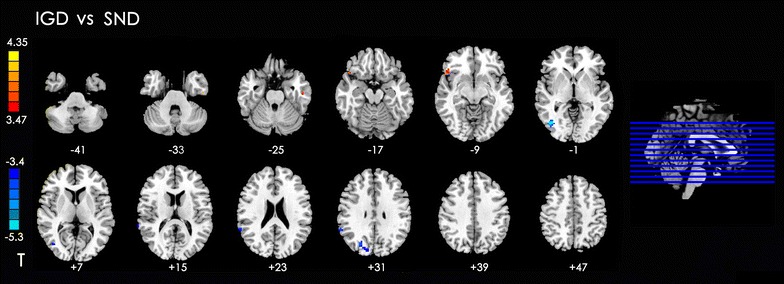
Significant between-group differences in functional connectivity of different brain regions with DLPFC between IGD and SND groups. The *t*-score bars are shown on the *left*. *Red* indicates IGD > SND, and *blue* indicates IGD < SND. *Note*: The *left part of figure* represents the participant’s right side, and *right* represents the participant’s left side. *DLPFC* dorsolateral prefrontal cortex, *IGD* internet gaming disorder, *SND* smokers with nicotine dependence

Compared with the SND group, the IGD subjects had increased rsFC in the left inferior temporal gyrus and right inferior orbital frontal gyrus and decreased rsFC in the right side of the middle occipital gyrus, supramarginal gyrus, and cuneus with the DLPFC (Table 5; Fig. 4).

**Table 5:** Summary of functional connectivity with DLPFC changes in IGD group compared with the SND group

	Peak MNI coordinate region	Peak MNI coordinates			Number of cluster voxels	Peak *t* value
		x	y	z		
1	Left inferior temporal gyrus (BA20)	−51	−15	−27	12	5.17
2	Right inferior orbital frontal gyrus (BA38)	48	27	−12	27	5.01
3	Right middle occipital gyrus (BA19)	42	−73	0	39	−6.70
4	Right supramarginal gyrus (BA48)	63	−48	27	26	−4.54
5	Right cuneus (BA19)	12	86	30	46	−4.58

*t* > 0 indicates IGD group >SND group in functional connectivity with DLPFC, *t* < 0 indicates IGD group <SND group in functional connectivity with DLPFC

*MNI* Montreal Neurological Institute, *BA* Brodmann’s area, *DLPFC* dorsolateral prefrontal cortex, *IGD* internet gaming disorder, *SND* smokers with nicotine dependence

*p* < 0.05, AlphaSim-corrected

#### Correlation between DLPFC connectivity and CIAS of IGD, DLPFC connectivity, and FTND of SND

Compared with the HC group, the IGD and SND both had decreased rsFC in the left inferior frontal gyrus and right insula with DLPFC. The rsFC strength values (mean zFC values) were extracted and averaged within a spherical ROI (radius of 10 mm) centered on the difference peak of the rsFC group (Tables 2, 3) in the IGD and SND groups. Pearson correlations were performed between the rsFC values with CIAS of the IGD group and the FTND score in SND group. However, no significant correlation was found.

## Discussion

In this study, we observe both similar and different brain connectivities in IGD group related to SND group. We detected that both the SND and IGD groups had decreased rsFC with DLPFC in the right insula and left inferior frontal gyrus. Furthermore, the IGD subjects exhibited different rsFC with DLPFC in the orbital frontal cortex and temporal, occipital, and parietal lobes.

Evidence revealed that many of the behavioral symptoms, even the neural mechanisms underlying IGD, resemble SUD [14, 34]. SUD involves a chronic, recurrent pattern of drug, nicotine, or alcohol use, and nicotine dependence is one of its most common forms. SUD could result in neurological alterations, particularly in frontal lobe structures implicated in cognitive-behavioral control. The network of cortical regions dysfunction, including the DLPFC, anterior cingulate cortex and lateral parietal cortex, relates to deficits in behavioral inhibition. This dysfunction has been linked to the loss of control over substance intake, which could be a critical step in the progression of SUD pathology [35, 36]. IGD is different from SUD in that no chemical or substance intake is involved; however, excessive internet use may also lead to physical dependence similar to that observed in other addictions [2]. Particularly, the hypo-activation of the inhibition circuit is a shared neural mechanism in SUD and behavioral addiction. Impaired function of the prefrontal cortex may relate to high impulsivity, which in turn, may contribute to impaired cognitive control and development of IGD [37]. Although the exact mechanism of IGD requires further investigation, its cognitive–behavioral model has been proposed. The model focuses on three domains including motivational drives related to reward-seeking and stress-reduction, behavioral control relating to executive inhibition, and decision-making that involves weighing the pros and cons of engaging in motivated behaviors [38].

Based on previous studies, both functional and structural abnormalities of the DLPFC have been commonly observed in IGD [39, 40]. Complex cognitive functions have usually been associated with activations in DLPFC [41] such as conflict-induced behavioral adjustment, attention, working memory, and inhibitory control [42–44]. DLPFC is connected with other cortical areas and links current sensory experiences to memory of past experiences to direct and generate properly goal-directed action [13, 45]. Therefore, the DLPFC may contribute to the coordination and keeping of the representations accepted from the other brain regions during the craving response when substance cues are present and a positive expectancy has been generated [46].

We detected that both the SND and IGD groups had decreased rsFC in the right insula and left inferior frontal gyrus with DLPFC. The insula has been implicated in cue-induced craving and relapse in nicotine-dependent tobacco cigarette smokers [47]. And the orbitofrontal cortex is involved in the evaluation of the reward of stimuli and explicit representation of reward expectancy for the substance [7]. Our results were consistent with the previous studies, which emphasized the brain regions, such as ventromedial prefrontal cortex, insula, thalamus, and cerebellum, which was critically linked with cigarette smoking. Structural MRI studies revealed that the integrities of the gray matters in the prefrontal cortex, anterior cingulate cortex, insula, thalamus, and cerebellum were reduced in smokers [48–50]. Liu et al. [51] investigated the brain function of IGD individuals using task-state fMRI. The IGD group showed increased activation in the right side of superior parietal lobule, insular lobe, precuneus, cingulated gyrus, superior temporal gyrus, and left side of brainstem. Internet video games activate the space, attention, vision, and execution centers located in the temporal, parietal, occipital, and frontal gyri. Abnormal brain function was noted in IGD subjects with hypofunction of the frontal cortex. Liu et al. detected IGD subjects that showed laterality activation of the right cerebral hemisphere, and they found that most areas were located in the right hemisphere. Neuroimaging studies in healthy subjects reported that the right hemisphere, especially in the right inferior frontal gyrus, is activated following successful response inhibition [52, 53]. During failed response inhibitions (i.e., trials that erroneously generated motor responses), the midline frontal structures, particularly the dorsomedial prefrontal cortex (dmPFC) encompassing pre-supplementary motor area and dorsal anterior cingulate cortex, are usually activated [54]. Consequently, the right inferior frontal gyrus is critical for response inhibition, whereas dmPFC is associate with response monitoring, particularly conflict and error monitoring [14].

The IGD subjects exhibited different rsFC with DLPFC in the orbital frontal cortex and temporal, occipital, and parietal lobes. Our result was partly similar with the result of a previous research compared rsFC with DLPFC in alcohol dependence with those in IGD [8]. They suggested that the connectivity observed in alcohol dependence is different from that in IGD because of the different comorbid diseases, early prevalence age, and visual and auditory stimulations in the former. Visual and auditory attentions are the results of the main sensory system inputs in response to internet game play [55]. Visual acuity loss or hearing problems may cause by extreme internet gaming [56]. Increased cortical volume within the parietal cortex was related to long-term gaming in pro-gamers, and thus may be related to increased visuospatial attention [57, 58].

Naturally, this study also comes with limitations. First, the cross-sectional design prevented us from determining whether the group differences in the rsFC are vulnerability factors for IGD and nicotine dependence. Second, the group sizes were unbalanced in our study, and the parameters such as sex, age, and education were not matched in the three groups. The unbalance group sizes might have influenced the results even though the variety was controlled during the statistical analysis. Third, the mean FTND in the SND group was 6.5, and thus the severity of nicotine dependence was not sufficiently high. Thus, increasing the number of participants is necessary.

## Conclusion

The rsFC is a very powerful tool for exploring multifaceted neuropsychiatric diseases, such as substance and non-substance addiction at system level. Our results confirmed that nicotine dependence and IGD may share similar mechanisms related to craving and impulsive inhibition. The observed difference between the rsFC of subjects with IGD and those of SND may be attributed to the impairments in audiovisual information processing by long-term internet gaming.

## Abbreviations

IGD: internet gaming disorder
SND: smokers with nicotine dependence
rsFC: resting-state functional connectivity
DLPFC: dorsolateral prefrontal cortex
HC: healthy controls
rs-fMRI: resting-state functional magnetic resonance imaging
PCC: post cingulate cortex
CIAS: Chen’s internet addiction score
AD: alcohol dependence
SUD: substance-related disorders
SAS: self-rating anxiety scale
SDS: self-rating depression scale
BIS-11: Barratt impulsiveness scale-11
FTND: Fagerstrom test of nicotine dependence
TR: repetition time
TE: echo time
FOV: field of view
FD: framewise displacement
ROI: region of interest
AFNI: Analysis of Functional Neuroimages
dmPFC: dorsomedial prefrontal cortex
